# MAX control: SUPPRESSOR OF MAX2 (SMAX)1-LIKE (SMXL) proteins repress growth in *Physcomitrium patens*

**DOI:** 10.1093/plcell/koae016

**Published:** 2024-01-23

**Authors:** Marco Bürger

**Affiliations:** Assistant Features Editor, The Plant Cell, American Society of Plant Biologists; Plant Biology Laboratory, Salk Institute for Biological Studies, La Jolla, CA 92037, USA

The KAI2 ligand (KL) is one or several unknown signaling molecule(s) involved in important plant processes, potentially representing an unidentified phytohormone. KL signaling, while interconnected with strigolactone (SL) signaling, distinctly influences plant development and photomorphogenesis. Both pathways utilize closely related receptor proteins, and in angiosperms, these receptors bind to the same F-box protein, MORE AXILLARY GROWTH 2 (MAX2), initiating the ubiquitination and subsequent degradation of SUPPRESSOR OF MAX2 (SMAX)1-LIKE (SMXL) proteins. Intriguingly, in the moss *Physcomitrium patens*, SL signaling is independent of MAX2 (Lopez-Obando et al. 2018), highlighting a unique aspect of this organism's biology. Previous research has explored multiple KL and SL receptors in *P. patens*, assigning them to their signaling pathways (Lopez-Obando et al. 2021). In a new study, Ambre Guillory and colleagues (Guillory et al. 2024) now demonstrate that SMXL proteins act as negative growth regulators in *P. patens*, functioning downstream of MAX2 in the KL signaling pathway.

Through phylogenetic analysis, Guillory and coworkers identified four distinct SMXL proteins, categorized into two clades (AB vs CD). They employed CRISPR-Cas9 mutagenesis to dissect the role of SMXL genes within the SL and KL pathways: mutants deficient in the AB clade mirrored the wild-type phenotype, while those lacking the CD clade exhibited enhanced growth, similar to the SL-deficient mutant *Ppccd8*. This pattern suggests that in the SL pathway, SMXL proteins may not function as repressors. The researchers then examined lines overexpressing certain SMXL genes. These lines displayed phenotypes resembling *Ppmax2-1* mutants, characterized by reduced radial growth, fewer but larger gametophores, and elongated gametophores under red light, reinforcing the concept of SMXL proteins as repressors within the MAX2-dependent pathway.

To further explore the connection between these proteins and the SL pathway, the team generated plants combining the SL-deficient *Ppccd8* background with *Ppsmxl* mutations. Strikingly, even a quintuple mutant, combining SL deficiency with a loss of all four *PpSMXL* genes, did not restore protonema extension to wild-type levels. This finding provided compelling evidence that PpSMXL proteins do not act as repressors in the SL pathway. In contrast, when investigating the role of these proteins in KL signaling by combining PpSMXL gene mutations with the *Ppmax2-1* mutant background, the authors observed a moderate correction of the *Ppmax2* mutant phenotypes. This observation supported the hypothesis that PpSMXL proteins, especially those from the CD clade, are functionally involved in the KL signaling pathway.

Guillory and colleagues then conducted pharmacological treatments with the KL mimic (−)-desmethyl-GR24 and the SL analog (+)-GR24 and assessed filament growth in response. All *Ppsmxl* double mutants reacted to the KL mimic, and *Ppsmxlcd* mutants showed a decreased response compared to wild type to both chemicals, implying an involvement of the CD clade in both KL and SL signaling. Finally, using fluorescence microscopy in *Nicotiana benthamiana* and *P. patens*, the researchers discovered that the SMXL proteins predominantly localize in the nucleus. Notably, these proteins were sensitive to possible destabilization after treatment with the KL mimic, but not with the SL analog.

These findings provide a deeper understanding of the molecular mechanisms underpinning moss growth regulation. The results support a model in which PpSMXL proteins are integral to the PpMAX2-dependent KL pathway (see Fig.) and provide hints at a possible interplay between SL and KL signaling. Given the independence of SL signaling from MAX2 in *P. patens*, PpSMXL proteins might play a crucial role in mediating the interaction between these two pathways in moss development.

**Figure.**
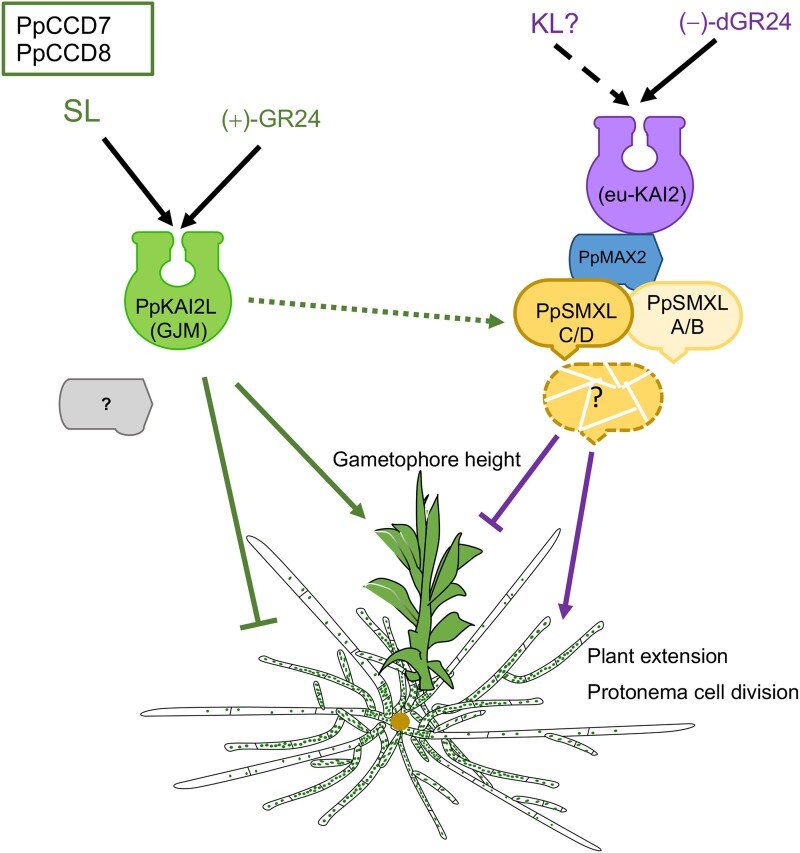
SL and KL signals have opposing effects on protonema extension and gametophore development in *P. patens*. Only the KL pathway depends on MAX2, with all four SMXL proteins likely acting as suppressors. Adapted from Guillory et al. (2024), Figure 7.

## Dive Curated Terms

The following phenotypic, genotypic, and functional terms are of significance to the work described in this paper:

kai2 Gramene: AT4G37470
kai2 Araport: AT4G37470
